# Safety of Postoperative Administration of Human Urinary Trypsin Inhibitor in Lung Cancer Patients with Idiopathic Pulmonary Fibrosis

**DOI:** 10.1371/journal.pone.0029053

**Published:** 2011-12-22

**Authors:** Yoshikane Yamauchi, Yotaro Izumi, Masanori Inoue, Hiroaki Sugiura, Taichiro Goto, Masaki Anraku, Takashi Ohtsuka, Mitsutomo Kohno, Kenzo Soejima, Hiroaki Nomori

**Affiliations:** 1 Department of Surgery, School of Medicine, Keio University, Tokyo, Japan; 2 Department of Diagnostic Radiology, School of Medicine, Keio University, Tokyo, Japan; 3 Department of Pulmonary Medicine, School of Medicine, Keio University, Tokyo, Japan; Institut Gustave Roussy, France

## Abstract

**Background:**

Patients with idiopathic pulmonary fibrosis (IPF) undergoing pulmonary resection for lung cancer carry risks of acute exacerbations of IPF (AE) postoperatively. Currently, agents which may attenuate AE are actively sought. Urinary trypsin inhibitor, ulinastatin, is a synthetic glycoprotein which may potentially inhibit various inflammatory factors associated with the development and progression of IPF. The present study was done to evaluate the effects of administration of high dose ulinastatin in lung cancer patients with IPF immediately following lung resection.

**Methods:**

Patients with IPFs radiologically diagnosed on high resolution CT, and histologically diagnosed resectable lung cancers, were eligible for the study. The effects of escalating doses of ulinastatin 3×10^5^, 6×10^5^, and 9×10^5^ units/body/day, administered postoperatively for 3 days were evaluated. The endpoints were safety and feasibility.

**Results:**

Nine patients were evaluated, in cohorts of 3 patients per dosage. Postoperative follow up ranged from 3 to 12 months (median 9 months). The postoperative courses were uneventful in all patients. No subjective adverse events such as abdominal symptoms or skin rashes, or objective adverse events as per serum laboratory tests, such as liver or kidney dysfunctions potentially attributable to ulinastatin administration were observed. AE was seen in one patient at 3 months after surgery, but since this occurred shortly after administration of chemotherapy, it was considered to be attributable to the chemotherapy rather than surgery.

**Discussion:**

Ulinastatin administration after lung resection in lung cancer patients with IPF was considered to be safe and feasible. Further study is planned at the highest dose of this study to evaluate efficacy.

**Trial Registration:**

UMIN.ac.jp/ctr/UMIN000002410

## Introduction

Idiopathic pulmonary fibrosis (IPF) is defined as a specific form of chronic, progressive fibrosing interstitial pneumonia of unknown cause, occurring primarily in older adults, limited to the lungs, and associated with histopathologic and/or radiologic pattern of usual interstitial pneumonia (UIP). Patients with IPF have been reported to have an increased risk of developing lung cancer compared with patients without IPF [1], [2], but there are also contradicting reports [3] and the evidence is currently conflicting.

IPFs are usually characterized by slowly progressive respiratory insufficiency. Nevertheless, some IPF patients experience acute exacerbations of IPF (AE) generally characterized by sudden onset of progressive, and severe respiratory failure, with rapid appearances of new lung opacities. This condition is frequently lethal since there is no established treatment for AE. According to a recent survey in Japan, the frequency of AE after surgical resection for lung cancer was reported to be 8.3%, and 41.9% of these patients died of AE [4].

Ulinastatin, or urinary trypsin inhibitor (Miracrid®, Mochida Pharmaceutical Co. Ltd., Tokyo, Japan) is a synthetic glycoprotein with a molecular weight of 67 kDa, first purified from human urine. It is frequently used clinically for the treatment of shock [5] and acute pancreatitis [6]. Ulinastatin is also known to inhibit various inflammatory factors associated with the development and progression of IPF, such as cytokines [7], oxygen radicals [8] and adhesion molecules [9]. In experimental studies, high dose, ulinastatin has been shown to have protective effects against radiation induced lung fibrosis in mice (2×10^5^ units/kg) [10], and rats (4×10^5^ units/kg) [11]. According to the pharmaceutical reference of ulinastatin, side effects have been reported in 74 out of 8710 patients (0.8%) at doses up to of 3×10^5^ units/day. These included abnormalities in serum tests such as elevations in liver enzymes, abdominal symptoms, skin rashes, and angialgias after intravenous administrations In clinical studies, administration of ulinastatin after lung resection for lung cancer has been shown to be safe at a dose of 3×10^5^ units/day for 4 days [12], [13]. It has also been reported that administration of high dose ulinastatin 9×10^5^ units/day resulted in clinical as well as radiological improvements of interstitial pneumonia in patients with connective tissue diseases [14], [15].

In view of these basic as well as clinical reports on the potential efficacy of ulinastatin on IPF, the present study was done to evaluate the effects of administration of high dose ulinastatin in lung cancer patients with IPF immediately following lung resection. The endpoints were safety and feasibility.

## Materials and Methods

### Patients

Patients between 20 and 80 years of age visiting our institution were considered for the study. The diagnosis of IPF was based on radiological findings on CT. Patients with IPF, and histologically diagnosed resectable lung cancers were eligible for the study. The diagnosis of IPF was made on a high resolution CT by two diagnostic radiologists certified by the Japan Radiological Society (M.I. and H.S.) with 13 and 15 years of clinical experiences, respectively. Diagnoses were made independently, and the two radiologists discussed when they turned out to be different. In the diagnostic criteria, the presence of honeycomb lung, subpleural predominance was necessary. At least one feature of tractional bronchitis, bronchiole ectasia, ground grass opacity, or consolidation was also necessary. The criteria are according to the general rules for Diagnosis and Treatment of Idiopathic Interstitial Pneumonias as described by The Japanese Respiratory Society [16]. The histology and tumor-node-metastasis classification were classified according to the general rules for clinical and pathologic recording of lung cancer as described by the Japan Lung Cancer Society [17].

Further inclusion criteria were a performance status of 0 or 1, adequate hematologic, hepatic, renal, and cardiac function diagnosed within 2 weeks before the registration, (white blood cells > = 3,000/mm^3^–< = 12,000/mm^3^, neutrophils count 1,500/mm^3^–5000/mm^3^, platelets count > = 75,000/mm^3^, hemoglobin > = 8.0 g/dL, aspartate aminotransferase and alanine aminotransferase under 2.5 times the upper institutional limit, total bilirubin <1.5 mg/dL, creatinine under 1.5 times the upper institutional limit, normal electrocardiogram, and SpO_2_>90% at room air), and being capable of tolerating general anesthesia.

The exclusion criteria were diagnoses of asbestosis, association with connective tissue diseases, chronic hypersensitivity pneumonitis, and sarcoidosis. Patients with documentations of allergic reactions against ulinastatin, or who were pregnant or lactating were also excluded.

### Study protocol

The study was carried out in the Division of General Thoracic Surgery, School of Medicine, Keio University, Japan, according to the standards of Good Clinical Practice for Trials on Medicinal Products in Japan. The protocol was approved by Institutional review board in Keio University School of medicine (no. 2009-73). Written informed consent was obtained from all of the patients before undergoing a screening evaluation to determine eligibility. (The protocol for this trial and supporting CONSORT checklist are available as supporting information; see Checklist S1 and Protocol S1.)

The primary endpoint was safety. Laboratory assessments including peripheral blood analyses, liver and kidney functions, were conducted on days 1, 3, 7, and 1 month after administration of ulinastatin. Any adverse events and changes in laboratory values were graded according to the National Cancer Institute Common Toxicity Criteria version 4.0. An independent data safety monitoring board (DSMB) consisting of three institutional members not involved in this study, one pneumologist, one radiologist, and one general surgeon, evaluated the data analyses, complications, and side effects. DSMB reviewed the data before the each case entered the trial. During the administration of ulinastatin, DSMB checked the data every day. After the administration, DSMB checked the data weekly until one month after surgery. The follow up period of this study was one month, but any latent side effects which were detected during subsequent patient follow up, which were suspected to be potentially attributable to ulinastatin administration were to be reported and evaluated.

All ulinastatin administrations were done in hospitalized patients. A predetermined dose of ulinastatin was administered 9 times per patient. Ulinastatin was intravenously administered as bolus injections, 2 milliliters per 10^5^ units, immediately after returning to the ward after surgery, and every 8 hours thereafter, as indicated for the treatment of shock. Three dose level cohorts were planned, 3×10^5^ units/body/day, 6×10^5^ units/body/day, and 9×10^5^ units/body/day. Three patients were to be entered for each dose cohort. Dose escalation was done after one-month-observation of the last patient in the previous cohort was complete. If any grade 4 toxicity was seen, the trial was to be stopped. If grade 3 toxicity was seen, the DSMB was to decide whether the trial could be continued or not, depending on the condition and causal relationship to ulinastatin administration.

The secondary endpoint was the incidence of AE. The diagnosis of AE was based on the fulfillment of all of the following criteria; 1) unexplained worsening or development of dyspnea within 30 days, 2) high-resolution computed tomography with new bilateral ground-glass abnormality and/or consolidation superimposed on a background reticular or honeycomb pattern consistent with usual interstitial pneumonia pattern 3) no evidence of pulmonary infection by endotracheal aspirate or bronchoalveolar lavage 4) exclusion of alternative causes, including left heart failure, pulmonary embolism, and other identifiable cause of acute lung injury [18].

## Results

### Study population

**Figure 1:**
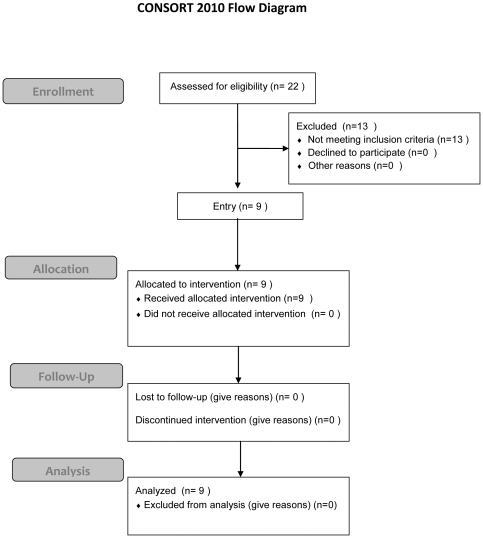
CONSORT flowchart of this trial.

This trial was conducted from September 2009 to April 2011. During this period, lung resections were performed in 125 patients with lung cancer. Of these patients, 22 patients were enrolled in the present study. Thirteen of these patients did not meet the radiological criteria for IPF and were excluded. Nine patients were included in the present study (Figure 1).

The demographic characteristics of the 9 patients are shown in Table 1. The patients were all males with smoking histories. Their ages ranged from 54 to 79 years (median 69 years). Performance status was 0 in all patients. Comorbidities included histories of other cancers, hypertension, diabetes, and aortic aneurysm, but were all sufficiently controlled. None of the patients were taking steroids, immunomodulators or pirfenidone. Lobectomy was done in 8 patients and segmentectomy was done in one patient. ND2a-II lymph node dissection (ipsilateral hilar and mediastinal lymph node dissection) was done in all patients. Radiological IPFs were seen predominantly in the lower lobes. UIP was seen histologically in patients No. 1, 2, 4, 7, 8, and 9, in which the region of resection included the radiological region of IPF, but was not apparent in other patients. The dosages of ulinastatin were 3×10^5^ units/body/day, 6×10^5^ units/body/day, and 9×10^5^ units/body/day, in patients No. 1 to 3, 4 to 6, and 7 to 9, respectively. Intravenous administration of ulinastatin was feasible in all patients.

**Table 1:** Patient demographics.

Patient No	1	2	3	4	5	6	7	8	9	Mean±SD, median
Age	73	59	79	69	69	67	54	66	75	68±8, 69
sex	M	M	M	M	M	M	M	M	M	-
Smoking (pack/years)	100	60	60	30	80	80	30	90	50	64±25, 60
PS	0	0	0	0	0	0	0	0	0	-
Comorbidities	diabetes	none	gastric Cancer, colon cancer, and renal cancer, all resected	abdominal aortic aneurysm, stent placed	oral cancer resected, gastric ulcer	laryngeal cancer, resected	hypertention	hypertention	hypertention, old pulmonary tuberculosis	-
Predominant region of IPF	bilateral lower lobes	bilateral lower lobes	bilateral lower lobes	bilateral lower lobes	bilateral lower lobes	bilateral lower lobes	bilateral lower lobes	all lobes	bilateral lower lobes	-
Operative procedure	left S6 segmentectomy	LLL	LUL	LLL	RUL	RUL	LLL	LUL	LLL	-
Histology	SCLC	SQ	SQ	SQ	LCNEC	AD	AD	SQ	AD	-
p-TNM	T1aN0M0	T3N0M0	T2bN0M0	T2aN2M0	T2N2M0	T2aN0M0	T3N0M0	T1bN0M0	T1bN0M0	-

M: male, PS: performance status, SCLC: small cell lung cancer, SQ: squamous cell carcinoma, LCNEC: large cell neuroendocrine carcinoma, AD: adenocarcinoma, LLL: left lower lobectomy, LUL: left upper lobectomy, RUL: right upper lobectomy, SD: standard deviation.

Perioperative factors, which may have potentially affected IPF status, are summarized in Table 2. Moderate variability existed in the perioperative factors among patients, but it was considered that operative procedures were completed without significant complications in all the patients.

**Table 2:** Perioperative factors that may have potentially affected interstitial pulmonary fibrosis status.

Patient No.	1	2	3	4	5	6	7	8	9	Mean±SD, median
Operative time (mins)	345	318	222	353	295	221	197	200	290	271±62, 290
Anesthesia time (mins)	449	393	317	455	430	295	261	320	355	364±71, 355
One lung ventilation time (mins)	360	320	187	350	310	194	187	186	265	262±75, 265
Intraoperative bleeding (ml)	90	177	90	170	277	50	47	133	132	130±72, 132
Total intraoperative oxygen (l)	890	1200	650	1800	900	885	550	960	880	968±362, 890

SD: standard deviation.

### Safety

Outcomes after surgery are summarized in Table 3. All patients were discharged without oxygen. Prolonged lung fistula was seen in patient No. 8, but otherwise the postoperative outcomes were uneventful. Symptomatic side effects potentially attributable to ulinastatin, such as abdominal symptoms or skin rashes were not seen in any of the patients. AE within one month was not seen in any of the patients (0/9, 0%, 95% confidence interval of the estimated population proportion 0–30%). AE was seen in patient No. 1 at 3 months after surgery, but since this occurred shortly after administration of chemotherapy, it was considered to be attributable to chemotherapy rather than surgery. This patient died of the AE 6 months later. Patient No. 2 died of cancer progression at 3 months after surgery. Since this patient had stage IIB disease, and furthermore, no postoperative chemotherapy was done due to IPF, this early progression was not considered as exceptional, and was not considered to be related to the administration of ulinastatin. Other patients are currently alive without disease.

**Table 3:** Postoperative outcomes.

Patient No.	1	2	3	4	5	6	7	8	9	Mean±SD, median
Nasal oxygen (days)	8	2	1	1	1	1	1	17	1	3.7±5.5, 1
Hospital stay (days)	11	5	10	10	10	9	8	21	11	10.6±4.3, 10
Postoperative complications	none	none	none	none	none	none	none	prolonged lung fistula	none	-
Symptomatic side effects potentially attributable to ulinastatin	none	none	none	none	none	none	none	none	none	-
AE within 1 month	none	none	none	none	none	none	none	none	none	-
Clinical outcome	AE at 3 POM after chemo-therapy. Died of AE at 9POM	Died of cancer at 3POM	Alive without disease at 1POY	Alive without disease at 9POM	Alive without disease at 9POM	Alive without disease at 9POM	Alive without disease at 6POM	Alive without disease at 3POM	Alive without disease at 3POM	-

POM: postoperative months, POY: postoperative years, SD: standard deviation.

**Figure 2:**
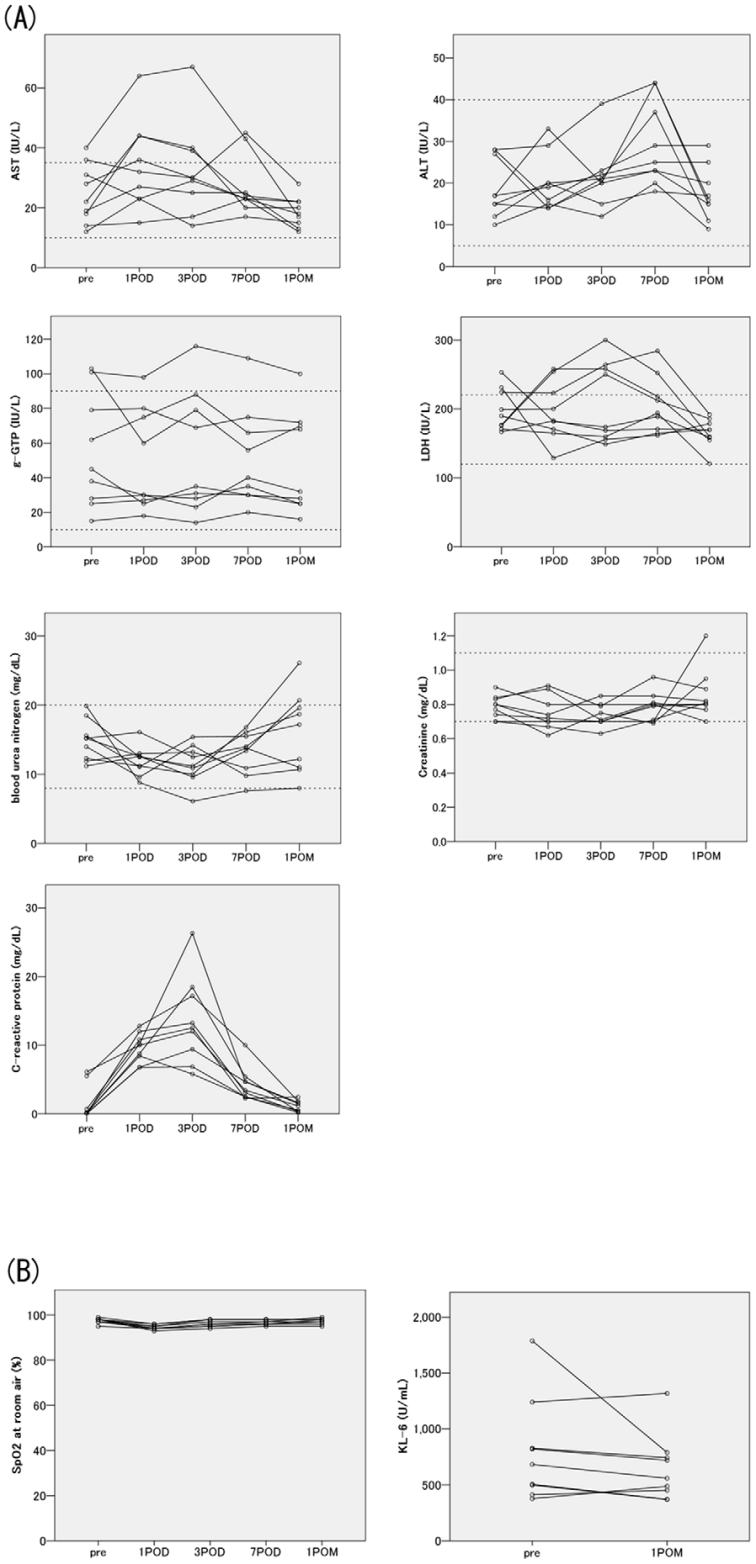
The postoperative changes in the laboratory data are shown. (A) In the serum laboratory data, the measurements shifted mostly within the institutional limits (dotted lines). The transient increases in c-reactive proteins were seen. POD: postoperative days, POM: postoperative months, AST: Asparatate Aminotransferase, ALT: Alanine Aminotransferase, LDH: Lactate Dehydrogenase, g-GTP: gamma-Glutamyltranspeptidase. (B) Arterial oxygen saturation at room air (SpO_2_), and sialylated carbohydrate antigen (KL-6) values, which were measured as indirect indices of IPF status, were mostly stable during the observation period.

Results of the serum blood test are summarized in Fig. 2A. The transient increases in c-reactive proteins were considered to be primarily due to surgery. Other measurements shifted mostly within the institutional limits, and side effects attributable to ulinastatin administration were not apparent. Arterial oxygen saturation at room air, and sialylated carbohydrate antigen values, which were measured as indirect indices of IPF status, were also mostly stable during the observation period (Fig. 2B).

**Figure 3:**
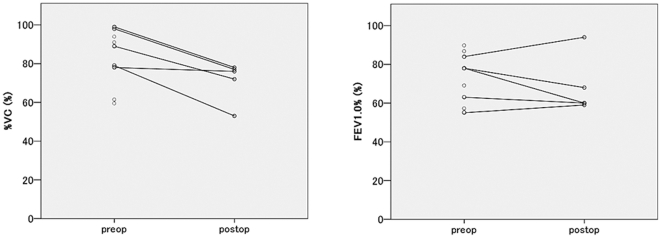
Postoperative pulmonary function tests were available in 5 patients. In these patients, both percent of predicted normal vital capacity (%VC) and percent of predicted normal forced expiratory volume in 1 second (FEV_1.0_%) decreased after lung resections.

Pulmonary function test results are summarized in Fig. 3. Postoperative pulmonary function tests were available in patients 5 to 9, and in these patients, the decreases in postoperative percent of predicted normal vital capacity and percent of predicted normal forced expiratory volume in 1 second were considered to be compatible with the performed lung resections.

## Discussion

Treatment for patients with lung cancer combined with IPF is problematic since idiopathic or iatrogenic AE could occur following various anticancer treatments. In the case of chemotherapy, the previously reported incidences of treatment-related AE ranged from 5.6% to 21% in Japan [19], [20]. The frequency of AE following conventional radiation therapy for IPF patients is reported to be around 25% in Japan [21], [22]. Therefore, conventional radiation therapy is considered to be contraindicated in IPF patients. The indication for stereotactic body radiotherapy still remains controversial [23]. Epidermal growth factor receptor tyrosine kinase inhibitors are also contraindicated in patients with IPF.

As for surgical resection, it has been reported that postoperative mortality and morbidity for major pulmonary resection is significantly higher in patients with IPF. Also, the long-term results tend to be poor due to the high incidence of second primary lung cancer as well as the poor natural history of IPF itself. Nevertheless, currently there is no evidence to indicate that surgical resection is contraindicated in lung cancer patients with IPF, particularly if the IPF is not considered to be rapidly progressive at the time of lung cancer diagnosis [24], [25], [26]. In view of these clinical data, we believe that the option of surgical resection should be offered to IPF patients with resectable lung cancer, if the patient understands and is willing to accept the risks involved.

Any treatment modality with a potential to attenuate AE should be actively sought. Treatment of AE has generally consisted of high-dose corticosteroids with empirical success in some patients, but there are no data from controlled trials to prove their efficacy [18], [27]. There are also no known agents which may provide prophylaxis for AE. Furthermore, there are no established predictive indices for AE [18]. Although the exact mechanisms are still to be elucidated, it has been reported that progression of IPF is associated with the production of factors such as MCP-1 (monocyte chemotactic protein-1) [28], and TGF-beta (transforming growth factor-beta) [10], [29]. Ulinastatin was first characterized as a protease inhibitor, but its effects seem to be multifaceted. Recently, it was suggested that serum concentration of both MCP-1 and TGF-beta were suppressed by administration of 9×10^5^ units of ulinastatin in patients with connective tissue disease associated pulmonary fibrosis [14], [15]. We considered that administration of ulinastatin at this dosage might decrease the incidence and/or severity of AE after lung resection. Since this dosage is three times the maximum approved dosage of ulinastatin in Japan, the present study was done to test the safety and feasibility of this dosage in lung cancer patients with IPF after lung resection.

In the present study, although the study population is small, ulinastatin administration after lung resection was considered to be safe in all patients. Its intravenous administration during the postoperative period was also considered to be feasible. Further accumulation of data at a dosage of 9×10^5^ units of ulinastatin is planned to evaluate efficacy. In future studies, it would be optimal to include a comparative control arm. But acquisition of patients' consents in such studies is expected be difficult because of the extremely poor outcome after exacerbation of IPF, and the relatively high safety profile of ulinastatin. A larger scale one-arm study may be more realistic.

## Supporting Information

Protocol S1 Trial Protocol.
(DOC)

Checklist S1 CONSORT Checklist.
(DOC)

## References

[1] Hubbard R, Venn A, Lewis S, Britton J (2000). Lung cancer and cryptogenic fibrosing alveolitis. A population-based cohort study.. Am J Respir Crit Care Med.

[2] Le Jeune I, Gribbin J, West J, Smith C, Cullinan P (2007). The incidence of cancer in patients with idiopathic pulmonary fibrosis and sarcoidosis in the UK.. Respir Med.

[3] Samet JM (2000). Does idiopathic pulmonary fibrosis increase lung cancer risk?. Am J Respir Crit Care Med.

[4] Miyamoto A, Kishi K, Yoshimura K (2011). [A nationwide survey concerning lung surgery for lung cancer associated with idiopathic interstitial pneumonia].. Nihon Kokyuki Gakkai Zasshi.

[5] Ohnishi H, Suzuki K, Niho T, Ito C, Yamaguchi K (1985). Protective effects of urinary trypsin inhibitor in experimental shock.. Jpn J Pharmacol.

[6] Ohnishi H, Kosuzume H, Ashida Y, Kato K, Honjo I (1984). Effects of urinary trypsin inhibitor on pancreatic enzymes and experimental acute pancreatitis.. Dig Dis Sci.

[7] Endo S, Inada K, Taki K, Hoshi S, Yoshida M (1990). Inhibitory effects of ulinastatin on the production of cytokines: implications for the prevention of septicemic shock.. Clin Ther.

[8] Cai M, Ogawa R (1994). Effects of free radical scavengers, methylprednisolone, and ulinastatin on acute xanthine and xanthine oxidase-induced lung injury in rats.. Circ Shock.

[9] Kawamura T, Inada K, Akasaka N, Wakusawa R (1996). Ulinastatin reduces elevation of cytokines and soluble adhesion molecules during cardiac surgery.. Can J Anaesth.

[10] Katoh H, Ishikawa H, Hasegawa M, Yoshida Y, Suzuki Y (2010). Protective effect of urinary trypsin inhibitor on the development of radiation-induced lung fibrosis in mice.. J Radiat Res (Tokyo).

[11] Bao P, Gao W, Li S, Zhang L, Qu S (2009). Effect of pretreatment with high-dose ulinastatin in preventing radiation-induced pulmonary injury in rats.. Eur J Pharmacol.

[12] Kondo D, Imaizumi M, Abe T (1993). [Study on extravascular lung water and polymorphonuclear leukocyte elastase during acute phase following radical treatment of lung cancer: effect of ulinastatin on respiratory functions].. Kyobu Geka.

[13] Sensaki K, Masuda H, Kikuchi K, Takagi K, Kase K (1993). Postoperative immunity in lung cancer patients and the effect of granulocyte protease inhibitor (ulinastatin).. Jpn J Lung Cancer.

[14] Tsujimura S, Saito K, Nakayamada S, Tanaka Y (2005). Human urinary trypsin inhibitor bolus infusion improved severe interstitial pneumonia in mixed connective tissue disease.. Mod Rheumatol.

[15] Tsujimura S, Saito K, Nakayamada S, Tanaka Y (2008). Bolus infusion of human urinary trypsin inhibitor improves intractable interstitial pneumonia in patients with connective tissue diseases.. Rheumatology (Oxford).

[16] The Japanese Respiratory Society (2004). Idiopathic Intestitial Pneumonias: Diagnosis and Treatment.

[17] The Japan Lung Cancer Society (2010). General rules for clinical and pathologic recording of lung cancer. 7th.ed.

[18] Collard HR, Moore BB, Flaherty KR, Brown KK, Kaner RJ (2007). Acute exacerbations of idiopathic pulmonary fibrosis.. Am J Respir Crit Care Med.

[19] Hoshikawa Y, Kondo T (2004). [Perioperative lung injury: acute exacerbation of idiopathic pulmonary fibrosis and acute interstitial pneumonia after pulmonary resection].. Nippon Geka Gakkai Zasshi.

[20] Minegishi Y, Sudoh J, Kuribayasi H, Mizutani H, Seike M (2011). The safety and efficacy of weekly paclitaxel in combination with carboplatin for advanced non-small cell lung cancer with idiopathic interstitial pneumonias.. Lung Cancer.

[21] Hanibuchi M, Yamaguchi T, Okada T, Nakagawa M, Yokota S (2001). Clinical examination of acute exacerbation of idiopathic intestitial Pneumonia (IIP) combined with lung cancer after anti-cancer treatment.. Jpn J Lung Cancer.

[22] Takenaka K, Yoshimura A, Okano T, Seike M, Kamio K (1999). Acute Exacerbation of idiopathic interstitial pneumonia complicated by lung cancer, caused by treatment for lung cancer.. Jpn J Lung Cancer.

[23] Takeda A, Enomoto T, Sanuki N, Nakajima T, Takeda T (2008). Acute exacerbation of subclinical idiopathic pulmonary fibrosis triggered by hypofractionated stereotactic body radiotherapy in a patient with primary lung cancer and slightly focal honeycombing.. Radiat Med.

[24] Fujimoto T, Okazaki T, Matsukura T, Hanawa T, Yamashita N (2003). Operation for lung cancer in patients with idiopathic pulmonary fibrosis: surgical contraindication?. Ann Thorac Surg.

[25] Kumar P, Goldstraw P, Yamada K, Nicholson AG, Wells AU (2003). Pulmonary fibrosis and lung cancer: risk and benefit analysis of pulmonary resection.. J Thorac Cardiovasc Surg.

[26] Watanabe A, Higami T, Ohori S, Koyanagi T, Nakashima S (2008). Is lung cancer resection indicated in patients with idiopathic pulmonary fibrosis?. J Thorac Cardiovasc Surg.

[27] Raghu G, Collard HR, Egan JJ, Martinez FJ, Behr J (2011). An Official ATS/ERS/JRS/ALAT Statement: Idiopathic Pulmonary Fibrosis: Evidence-based Guidelines for Diagnosis and Management.. Am J Respir Crit Care Med.

[28] Ebina M, Taniguchi H, Miyasho T, Yamada S, Shibata N (2011). Gradual increase of high mobility group protein b1 in the lungs after the onset of acute exacerbation of idiopathic pulmonary fibrosis.. Pulm Med.

[29] Willis BC, Liebler JM, Luby-Phelps K, Nicholson AG, Crandall ED (2005). Induction of epithelial-mesenchymal transition in alveolar epithelial cells by transforming growth factor-beta1: potential role in idiopathic pulmonary fibrosis.. Am J Pathol.

