# Supplementary material for: Safety of Postoperative Administration of Human Urinary Trypsin Inhibitor in Lung Cancer Patients with Idiopathic Pulmonary Fibrosis
Source: PLoS One. 2011 Dec 22;6(12):e29053. doi: 10.1371/journal.pone.0029053 (PMC3245251; doi:10.1371/journal.pone.0029053)
Supplement: Protocol S1 — Trial Protocol. (DOC) [file pone.0029053.s001.doc]

# STUDY PROTOCOL

**Phase I study for the safety of the postoperative ulinastatin in patients with resectable lung cancer accompanied with interstitial pneumonitis**

This was translated from Japanese original protocol, which was created on August 14, 2009

**Principal investigator**

Yotaro Izumi

Division of General Thoracic Surgery,Department of Surgery, School of Medicine Keio, University

35 Shinanomachi, Shinjuku-ku, Tokyo 160-8582, Japan

Phone: +81-3-5363-3806 Fax: +81-3-5363-3499

E-mail: yotaroizumi@a2.keio.jp

**Clinical investigator**

Yoshikane Yamauchi

Hiroaki Nomori

Division of General Thoracic Surgery,Department of Surgery, School of Medicine Keio, University

35 Shinanomachi, Shinjuku-ku, Tokyo 160-8582, Japan

Phone: +81-3-5363-3806 Fax: +81-3-5363-3499

**Registry**

UMIN CTR (<http://www.umin.ac.jp/ctr>)

ID: UMIN000002410

**TABLE OF CONTENTS**

**1.0 INTRODUCTION**

**2.0 STUDY OBJECTIVES**

**3.0 TRIAL DESIGN**

**3.1 Main outcome measures**

**3.2 Study Design**

**3.3 Measures to Minimize bias**

**3.4 Study Product**

**3.5 Duration of the Study**

**4.0 SELECTION OP PARTICIPANTS**

**5.0 ADVERSE EVENTS**

**5.1 Definition**

**5.2 Evaluation of Adverse Events**

**6.0 ETHICS AND RESEARCH INTEGRITY**

**6.1 Fundamental principle**

**6.2 Institutional Review Board**

**6.3 Ensuring Human-rights**

**7.0 REFERENCES**

**1.0 INTRODUCTION**

Idiopathic pulmonary fibrosis (IPF) is defined as a specific form of chronic, progressive fibrosing interstitial pneumonia of unknown cause, occurring primarily in older adults. Patients with IPF have been reported to have an increased risk of developing lung cancer compared with patients without IPF^1,2^.

Lung resections were often performed in IPF patients with resectable lung cancer. Nevertheless, some patients experience acute exacerbations of IPF (AE) generally characterized by sudden onset of progressive, and severe respiratory failure, with rapid appearances of new lung opacities. This condition is frequently lethal since there is no established treatment for AE. According to a recent survey in Japan, the frequency of AE after surgical resection for lung cancer was reported to be 8.3 %, and 41.9% of these patients died of AE. Therefore it is required how to prevent or to avoid the AE.

Causes of AE have not been revealed yet, although the exposure to the high concentration of oxygen under general anesthesia, surgical stress, viral infection and etc. is considered to be candidates. Idiopathic pulmonary fibrosis (IPF) is characterized by the accumulation of inflammatory cells in the airspaces and a subsequent fibrosis of alveolar walls and the interstitium, which cytokines’ imbalance would be involved. Similar to AE, acute respiratory distress syndrom (ARDS) makes lung fibrosis subsequently. In the mechanism of lung dysfunction caused by ARDS, nuetrophil activated by various triggers emit the tissue-damagable mediators, causing lung damages.

Ulinastatin, or urinary trypsin inhibitor (Miracrid®, Mochida Pharmaceutical Co. Ltd., Tokyo, Japan) is a synthetic glycoprotein with 143 amino acids and 2 glycans, first purified from human urine. According to the pharmaceutical reference of ulinastatin, side effects have been reported in 74 out of 8710 patients (0.8%) at doses up to of 3 × 10^5^ units/day. These included abnormalities in serum tests such as elevations in liver enzymes, abdominal symptoms, skin rashes, and angialgias after intravenous administrations. Ulinastatin is also known to inhibit various inflammatory factors associated with the development and progression of IPF, such as cytokines^3^, oxygen radicals^4^ and adhesion molecules^5^. In experimental studies, high dose, ulinastatin has been shown to have protective effects against radiation induced lung fibrosis in mice (2× 10^5^ units/kg)^6^, and rats (4× 10^5^ units/kg)^7^. In clinical studies, administration of ulinastatin after lung resection for lung cancer has been shown to be safe at a dose of 3 × 10^5^ units/day for 4 days^8^. It has also been reported that administration of high dose ulinastatin 9 × 10^5^ units/day resulted in clinical as well as radiological improvements of interstitial pneumonia in patients with connective tissue diseases^9,10^.

In the phase I clinical trial for patients with resectable lung cancer accompanied with interstitial pneumonitis by postoperative administration of ulinastatin, neither adverse reactions nor prevention of AE were observed.

**2.0 STUDY OBJECTIVES**

The purpose of this clinical trial is to both analyse the safety and the efficacy of postoperative administration of ulinastatin for patients with resectable lung cancer accompanied with interstitial pneumonitis for the purpose of prevention of postoperative AE.

**3.0 TRIAL DESIGN**

**3.1 Main outcome measures**

(Primary outcomes)

We examined whether and to what extent adverse events, for which causal relationships with the administration of ulinastatin were not ruled out, emerged. The measurement of the evaluation for adverse events was the grade according to NCI-CTCAE v4.0. An independent data safety monitoring board (DSMB) consisting of three institutional members not involved in this study, one pneumonologist, one radiologist, and one general surgeon, evaluated the data analyses, complications, and side effects.

(Secondary outcomes)

We examined whether postoperative AE was observed in any of the patients or not. The diagnosis of AE was based on the fulfillment of all of the following criteria; 1) unexplained worsening or development of dyspnea within 30 days, 2) high-resolution computed tomography with new bilateral ground-glass abnormality and/or consolidation superimposed on a background reticular or honeycomb pattern consistent with usual interstitial pneumonia pattern 3) no evidence of pulmonary infection by endotracheal aspirate or bronchoalveolar lavage 4) exclusion of alternative causes, including left heart failure, pulmonary embolism, and other identifiable cause of acute lung injury. This diagnosis was judged by DSMB. Further evaluation was performed on the decline of SpO_2_ and blood test result.

**3.2 Study Design**

This study was single-armed, non-randomized, and non-blinded clinical study.

All ulinastatin administrations were done inpatient. A predetermined dose of ulinastatin was administered 9 times per patient. Ulinastatin was intravenously administered as bolus injections, 2 milliliters per 10^5^ units, immediately after returning to the ward after surgery, and every 8 hours thereafter, as indicated for the treatment of shock. Three dose level cohorts were planned, 3×10^5^ units/body/day, 6×10^5^ units/body/day, and 9×10^5^ units/body/day. Three patients were to be entered for each dose cohort. Dose escalation was done after one-month-observation of the last patient in the previous cohort was complete. Laboratory assessments including peripheral blood analyses, liver and kidney functions, were conducted on days 1, 3, 7, and 1 month after administration of ulinastatin. If any grade 4 toxicity was seen, the trial was to be stopped. If grade 3 toxicity was seen, the DSMB was to decide whether the trial could be continued or not, depending on the condition and causal relationship to ulinastatin administration.

**3.3 Measures to Minimize bias**

Many factors, such as smoking history, sex, or activity of IPF would influence on the study result. However, no factor has benn defined as an influential factor.

**3.4 Study Product**

Ulinastatin, or urinary trypsin inhibitor (Miracrid®) was purchased from Mochida Pharmaceutical Co. Ltd., Tokyo, Japan. The concentration was 2 milliliters per 10^5^ units.

**3.5 Duration of the Study**

This clinical trial including recruitment of subjects will be performed from September 1^st^, 2009 to August 31^st^, 2011.

**4.0 SELECTION OP PARTICIPANTS**

IPF Patients with lung caner visiting Keio University hospital will be requested to participate.

In compliance with Good Clinical Practice guidelines, prospective patients who provide written informed consent will undergo clinical inspection. We will then select as subjects 9 patients who satisfy the selection and exclusion criteria described below.

We selected those patients who met the criteria listed below,

<inclusion criteria>

1) Histologically confirmed lung cancer

2) cStage IA-IIIB, resectable

3) lung resection is planned

4) the case diagnosed as lung cancer with interstitial pneumonitis by one clinical radiologist in our hospital. The standard of diagnose is below.

1.coin lesion just under the pleura

2.honeycomb lung

3.tractional bronchitis, bronchioles ectasia

4.ground grass opacity

5.consolidation

5)performance status of 0 to 1

6)Adequate hematologic, hepatic, renal, and cardiac function diagnosed within 2 weeks before the registration.

*WBC >=3,000/mm3 - <=12,000/mm3

*Absolute neutrophil count 1,500/mm3 - 5000/mm3

*Platelet count >=75,000/mm3

*Hemoglobin >=8.0g/dL

*AST and ALT 2.5 times upper limit of normal

*Total bilirubin <1.5mg/dL

*Creatinine 1.5 times upper limit of normal

*ECG normal

*SpO2 90% (room air)

7) general anesthesia is permitted

8) Written informed consent

<exclusion criteria>

a)asbestosis

b)Patients with interstitial pneumonitis caused by connective tissue disease

c)chronic hypersensitivity pneumonitis

d)sarcoidosis

e)Patients who have the history of allergic reactions against urinastatin

f)Patients who are pregnant or have possibility of pregnancy

**5.0 ADVERSE EVENTS**

**5.1 Definition**

An adverse event is any unfavorable and unintended sign (including an abnormal laboratory finding, for example), symptom, or disease temporally associated with the use of ulinastatin, whether or not considered related to ulinastatin.

**5.2 Evaluation of Adverse Events**

The investigators must report any adverse event with the exact name, the onset date, the severity, the provided treatment and the date of disappearance.

Each adverse event should be graded for severity using the following scale and the criteria notified by NCI-CTCAE v4.0. DSMB must determine the relationship of the adverse event to ulinastatin. DSMB must determine whether or not the adverse event is classified as a serious adverse event. A serious adverse event is defined as Grade-3 or more by NCI-CTCAE v4.0.

**6.0 ETHICS AND RESEARCH INTEGRITY**

**6.1 Fundamental principle**

We will perform this trial in compliance with ICH Harmonised Tripartite Guideline E6.

**6.2 Institutional Review Board**

Prior to this clinical trial, this study must be approved in writing by the Institutional Review Board at each facility. The facilities will obtain written reapproval of the research by the IRBs at least annually.

**6.3 Ensuring Human-rights**

We comply with Declaration of Helsinki and place maximum priority on ensuring human-rights, welfare and safety of the subjects.

**7.0 REFERENCES**

1. Hubbard, R., Venn, A., Lewis, S. & Britton, J. Lung cancer and cryptogenic fibrosing alveolitis. A population-based cohort study. *Am J Respir Crit Care Med* **161**, 5-8 (2000).

2. Le Jeune, I.*, et al.* The incidence of cancer in patients with idiopathic pulmonary fibrosis and sarcoidosis in the UK. *Respir Med* **101**, 2534-2540 (2007).

3. Endo, S., Inada, K., Taki, K., Hoshi, S. & Yoshida, M. Inhibitory effects of ulinastatin on the production of cytokines: implications for the prevention of septicemic shock. *Clin Ther* **12**, 323-326 (1990).

4. Cai, M. & Ogawa, R. Effects of free radical scavengers, methylprednisolone, and ulinastatin on acute xanthine and xanthine oxidase-induced lung injury in rats. *Circ Shock* **43**, 71-78 (1994).

5. Kawamura, T., Inada, K., Akasaka, N. & Wakusawa, R. Ulinastatin reduces elevation of cytokines and soluble adhesion molecules during cardiac surgery. *Can J Anaesth* **43**, 456-460 (1996).

6. Bao, P.*, et al.* Effect of pretreatment with high-dose ulinastatin in preventing radiation-induced pulmonary injury in rats. *Eur J Pharmacol* **603**, 114-119 (2009).

7. Kondo, D., Imaizumi, M. & Abe, T. [Study on extravascular lung water and polymorphonuclear leukocyte elastase during acute phase following radical treatment of lung cancer: effect of ulinastatin on respiratory functions]. *Kyobu Geka* **46**, 922-925 (1993).

8. Sensaki K, *et al*. Postoperative immunity in lung cancer patients and the effect of granulocyte protease inhibitor (ulinastatin). Jpn J Lung Cancer **33**, 61-69 (1993).

9. Tsujimura, S., Saito, K., Nakayamada, S. & Tanaka, Y. Human urinary trypsin inhibitor bolus infusion improved severe interstitial pneumonia in mixed connective tissue disease. *Mod Rheumatol* **15**, 374-380 (2005).

10. Tsujimura, S., Saito, K., Nakayamada, S. & Tanaka, Y. Bolus infusion of human urinary trypsin inhibitor improves intractable interstitial pneumonia in patients with connective tissue diseases. *Rheumatology (Oxford)* **47**, 907-913 (2008).
